# MAFG Induces the Methylation of CRYAB to Promote the Activation of A1 Astrocyte After Spinal Cord Injury

**DOI:** 10.1002/iid3.70334

**Published:** 2026-01-19

**Authors:** Xuefei Li, Zhuang Zhu, Ying Wang, Hao Wan, Zhiwei Wang, Wanqing Qiao

**Affiliations:** ^1^ Department of Orthopedics The Second Affiliated Hospital of Shandong First Medical University Tai'an Shandong China; ^2^ The Second Affiliated Hospital of Shandong First Medical University Tai'an Shandong China

**Keywords:** A1/A2 astrocytes, CRYAB, inflammatory cytokines, MAFG, spinal cord injury

## Abstract

**Purpose:**

To investigate the effects of MAF bZIP transcription factor G (MAFG) on the transformation of A1/A2 reactive astrocytes and the production of inflammatory factors after spinal cord injury (SCI).

**Methods:**

An SCI model was established using Sprague–Dawley rats. Astrocyte conditioned medium (ACM) and lipopolysaccharide (LPS) were used to induce the generation of type A1 astrocytes. MAFG‐, CRYAB‐, C3‐, and S100A10‐positive cells were examined using immunofluorescence. The expression of MAFG, TNF‐α, IL‐1β, IL‐6, C3, Serping1, Sphk1, S100A10, CRYAB, DNMT1, DNMT3a, and DNMT3b was detected through RT‐PCR and/or Western blot. The inclined plate test and Basso‐Beattie‐Bresnahan scores were used to evaluate the motor function in rats. Hematoxylin and eosin and Nissl staining were performed to assess pathological changes in the rat spinal tissues. In rat astrocytes, IL‐1β and IL‐6 levels were examined via enzyme‐linked immunosorbent assay.

**Results:**

A1 astrocyte activation was accompanied by MAFG upregulation in rat spinal cord tissues after SCI. MAFG silencing inhibited the activation of A1 astrocytes and inflammation and improved neurological outcomes and functional recovery in rats after SCI. In ACM‐treated rat astrocytes, MAFG silencing inhibited A1 astrocyte activation, inflammation, and CRYAB methylation. Moreover, 5‐Aza (an inhibitor of methylation) further inhibited the activation of A1 astrocytes and inflammation, whereas DNMT3b overexpression had the opposite effect.

**Conclusion:**

Silencing MAFG reduced the activation of A1 astrocytes and neuroinflammation and improved functional recovery after SCI, which might be involved in the inhibition of CRYAB methylation.

## Summary

- In SCI tissues, A1 astrocyte polarization is accompanied by MAFG upregulation.
- MAFG silencing promotes rat functional recovery after SCI.
- MAFG silencing inhibits A1 astrocyte polarization and neuroinflammation.
- MAFG silencing inhibits the methylation of CRYAB.

## Graphical abstract

All in all, silencing MAFG inhibited the activation of A1 astrocyte and neuroinflammation through regulating CRYAB methylation.

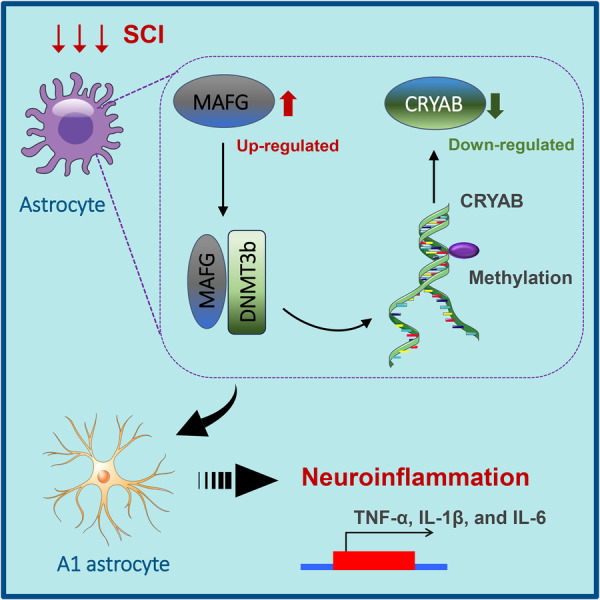

## 1 Introduction

As a form of central nervous system (CNS) trauma, spinal cord injury (SCI) leads to motor, sensory, and autonomic functional deficits, significantly impacting patients' quality of life and imposing a substantial burden on their families and society [1, 2]. Globally, the incidence of SCI is high at approximately 250,000–500,000 people 1 year [2]. Although SCI mortality has been reduced by the rapid provision of specialized medical and surgical care, the therapeutic effect on SCI is currently unsatisfactory [3, 4]. Primary and delayed secondary injuries are the two phases of SCI [5]. Primary injury is caused by precipitating events (e.g., road accidents, violence, ischemia, falls, and trauma) [6]. Secondary injury is a gradual process that involves a series of physiological and biochemical reactions such as neuroinflammation, edema, hematoma, and diffuse apoptosis of astrocytes [7, 8]. Therefore, a better understanding of the cellular and molecular changes that occur after injury is important for SCI treatment.

MAFG is a small v‐maf avian musculoaponeurotic fibrosarcoma oncogene homolog (MAF) protein [9]. According to the report, MAFG can form homodimers and inhibit the expression of antioxidant response element [10, 11]. MAFG is highly expressed in the nervous system, lymph nodes, skeletal muscles, lungs, osteosarcoma, and thyroid tissues [12, 13, 14]. Yang et al. indicated that MAFG expression was upregulated in mice treated with bile duct ligation (BDL) and that MAFG knockdown protected against liver injury after BDL [15]. Wheeler et al. suggested that stimulation of IL‐1β and TNF increased MAFG expression in primary mouse astrocytes [16]. However, the role of MAFG in SCI has not been investigated.

MAFG mediates many types of DNA methylation, including that of CRYAB [16]. CRYAB, also known as HspB5 or αB‐Crystallin, is a key member of small heat‐shock protein [17]. CRYAB is expressed in the lenses of the eye, ovaries, and skeletal muscle [18, 19, 20]. Previous studies have reported that CRYAB inhibits cell death and oxidative stress and promotes angiogenesis [21, 22, 23]. In a colitis model of mice, CRYAB overexpression suppressed the production of pro‐inflammatory cytokines, such as TNF‐α, IL‐6, and IL‐1β [24]. Knockdown of CRYAB increased inflammation in mice with experimental autoimmune encephalomyelitis [25]. Furthermore, CRYAB was reported to inhibit inflammatory responses in astrocytes [26, 27]. Based on this, we suspected that MAFG plays a role in SCI and may be related to CRYAB.

In this study, we assessed the changes and potential functions of MAFG in animal models of SCI and examined its possible mechanisms. Our findings suggest a potential therapeutic intervention for SCI.

## 2 Materials and Methods

### 2.1 Bioinformatic Analysis

The Gene Expression Omnibus (GEO) database GSE132242 was used to analyze the differential expression of MAFG. In the GSE132242 dataset, total RNA extracted from the T9‐T10 spinal cord segments of SCI and sham mice was analyzed.

### 2.2 Animals

The Animal Care and Use Committee of the Second Affiliated Hospital of Shandong First Medical University approved this study. Jinan Pengyue Experimental Animal Breeding Co. Ltd. (China) provided male Sprague–Dawley (SD) rats (8 weeks, 220–250 g). Under a 12‐h light‐dark cycle, all rats could drink and eat freely in humidity 60 ± 10% at 22 ± 2°C.

### 2.3 Construction of the SCI Animal Model

The rats were randomized into the sham, SCI, SCI + LV‐shNC, and SCI + LV‐shMAFG groups (*n* = 6 per group). To construct the SCI model, the rats were first intraperitoneally injected with pentobarbital sodium (50 mg/kg; Sigma‐Aldrich, USA) for anesthesia. Next, to expose the spinal cord, laminectomy was performed on the T10 segment. The exposed spinal cord segment was struck using a weight‐drop device (New York University, NY, USA), and the muscles and skin were quickly sutured. Following surgery, the rat bladders were squeezed manually twice daily until spontaneous micturition resumed. In the sham group, the spinal cord segment was exposed but no injury was observed. In the SCI + LV‐shNC and SCI + LV‐shMAFG groups, rats were injected in situ with 1.0 × 10^9^ TU/mL lentiviruses (LV) (Tsingke, Beijing, China) containing shRNA negative control (shNC; 5′‐ GCGTGATCTTCACCGACAAGATTCAAGAGATCTTGTCGGTGAAGATCACGCTTTTTT‐3') or MAFG specific shRNA (shMAFG; 5′‐ GCATGAAGCTGGAGCTCGATGTTCAAGAGACATCGA GCTCCAGCTTCATGCTTTTTT‐3′) after surgery, respectively. After surgery for 28 days, under deep anesthesia by pentobarbital sodium, rats were sacrificed. After transcardial perfusion, the spinal cord tissues were dissected.

### 2.4 Isolation of Rat Primary Astrocyte

Primary rat astrocytes were obtained from SD rats (1–2 days old). Briefly, the spinal cord tissues of the rats were cut into pieces. Next, tissue pieces were digested with 0.25% trypsin (Solarbio Life Sciences, China) for 10 min at 37°C and then incubated with DMEM/F12 medium (Gibco, USA) containing with 10% fetal bovine serum (FBS, Gibco). After centrifuging for 5 min at 300 *g*, the tissue pellet was re‐suspended in DMEM/F12 medium and then passed through the 100 µm nylon mesh to obtain a single‐cell suspension. Single‐cell suspensions were cultured in T75 flasks pre‐coated with poly‐l‐lysine (Sigma‐Aldrich). After culture for 12–14 days, non‐astrocytes were removed by shaking and changing the medium. Astrocytes were confirmed by positive staining for the astrocytic marker glial fibrillary acid protein (GFAP). The third‐generation astrocytes were used for further experiments.

### 2.5 Cell Transfection and Treatment

To stimulate the production of A1 type astrocytes, rat astrocytes were treated with astrocyte conditional medium (ACM: DMEM supplemented with 30 ng/mL TNF‐α, 3 ng/mL IL‐1α, and 400 ng/mL C1q) or 100 ng/mL lipopolysaccharide (LPS) for 24 h. In ACM + siNC, ACM + siMAFG, LPS + siNC, LPS + siMAFG, ACM + vector, and ACM + CRYAB group, cells were transfected with siRNA negative control (siNC; 5′‐GACAAAGAAACCCGACAAACT‐3′), MAFG specific siRNA (siMAFG; 5′‐GACCCCCAATAAAGGAAACAA‐3′), vector or CRYAB overexpression plasmid (Beijing Tsingke Biotech Co. Ltd.) using Lipofectamine 2000 Reagent (Invitrogen, USA). After transfection for 48 h, cells were treated with ACM or LPS. In ACM + siMAFG + DNMT3b group, cells were transfected with siMAFG and DNMT3b overexpression plasmid (Beijing Tsingke Biotech Co. Ltd.) using Lipofectamine 2000 Reagent, after transfection for 48 h, cells were treated with ACM. To inhibit DNA methylation, in ACM + siMAFG + 5‐Aza group, cells were transfected with siMAFG using Lipofectamine 2000 Reagent, after transfection for 48 h, cells were treated with 1 µmol/L 5‐azacitidine (5‐Aza, Sigma‐Aldrich) for 1 h before A1 astrocyte induction.

### 2.6 Behavioral Analysis

To evaluate the recovery of motor function in the rats, the Basso–Beattie–Bresnahan (BBB) functional score (21‐point system: 0, complete paralysis; 21, complete mobility) and inclined plate tests were performed on the rats at 0, 1, 3, 7, 14, 21, and 28 days after surgery. The BBB scoring indicators included the joint activity of the hindlimbs, gait of the hindlimbs, coordination of the hindlimbs, and fine movements of the claws. For the inclined plate test, the rats were placed on an inclined plane with a rubberized surface. Subsequently, one end of the inclined plane is gradually increased to increase the tilt angle. The maximum angle at which the rat could remain on the inclined plate for 5 s was recorded. Each rat was measured five times, and the average value was considered as the measured value.

### 2.7 Hematoxylin and Eosin (H&E) Staining

Spinal cord tissues were placed in 4% paraformaldehyde (Thermo Fisher Scientific) overnight and embedded in paraffin. Next, tissue samples were sliced into 5 μm‐thick sections. After warming at 60°C for 4 h, sections were treated with xylene (Sigma‐Aldrich) for 10 min and different gradients of ethanol (100%, 5 min; 90%, 2 min; 80%, 2 min; 70%, 2 min). Sections were stained with hematoxylin (Beyotime Biotechnology) for 5 min and eosin (Beyotime Biotechnology) for 2 min. Finally, the sections were dehydrated using different gradients of ethanol (70%, 10 s; 80%, 10 s; 90%, 10 s; and 100%, 10 s), dewaxed with xylene for 5 min, and sealed with neutral gum. Pathological changes were observed under a light microscope (Olympus).

### 2.8 Nissl Staining

After dewaxing and hydration, the tissue sections were placed in 1% toluidine blue solution (Solarbio Life Sciences) for 15 min. Subsequently, the sections were treated with 95% ethanol for 5 min, dehydrated with 100% ethanol, and cleared using xylene. After assembly with neutral gum, the tissue sections were observed under a microscope.

### 2.9 Immunofluorescence

For rat astrocytes, after the indicated treatment, the cells were placed in 4% paraformaldehyde for 15 min. After blocking with 10% goat serum for 1 h, cells were incubated with primary antibodies against MAFG, CRYAB (Abcam); C3, S100A10 (Proteintech, China) at 4°C overnight, maintained with secondary antibodies for 60 min, and treated with 4′,6‐diamidino‐2‐phenylindole (DAPI, Sigma‐Aldrich) for 10 min. After dewaxing, hydration, and antigen retrieval, the spinal cord tissue sections were incubated with primary antibodies against MAFG, C3, and S100A10 at 4°C overnight and treated with secondary antibodies for 1 h. Cells in the tissues were also stained. Positive cells were observed under a fluorescence microscope (Olympus).

### 2.10 Reverse Transcription‐Polymerase Chain Reaction (RT‐PCR)

Total RNA was isolated from the spinal cord tissues and rat astrocytes using TRIzol reagent (Invitrogen). Evo M‐MLV RT Premix (Accurate Biotechnology) was used for cDNA synthesis. RT‐PCR was performed using the Power SYBR Green PCR Premix (Thermo Fisher Scientific). Relative mRNA expression was analyzed by the 2^−ΔΔCt^ method. Shanghai Sangon Biotech Co. Ltd. (China) provided all primers, the primer sequences were listed as follows: MAFG [forward (F): 5′‐CAAGGCCTTAAAGGTGAAGCG‐3′, reverse (R): 5′‐ GCTCCTCCTTCTGTGTCACC‐3′)], C3 (F: 5′‐AGAACTGGTCAACATGGGGC‐3′, R: 5′‐TCCCCTGAACCATCCTCGAT‐3′), Serping1 (F: 5′‐CCCTGAAGCTGCCTAGTGAC‐3′, R: 5′‐AGAAGGCTCTATCCCCAGCTA‐3′), S100A10 (F: 5′‐ATCCCAAATGGAGCATGCCA‐3′, R: 5′‐CCAGAGGGTCCTTTTGATTTTCC‐3′), Sphk1 (F: 5′‐TTCCGAGCAGAAAGGGAACC‐3′, R: 5′‐TTAGCCCATTCACCACCTCG‐3′), TNF‐α (F: 5′‐ATGGGCTCCCTCTCATCAGT‐3′, R: 5′‐GCTTGGTGGTTTGCTACGAC‐3′), IL‐1β (F: 5′‐GGGCCTCAAGGGGAAGAATC‐3′, R: 5′‐TTTGGGATCCACACTCTCCAG‐3′), IL‐6 (F: 5′‐AGAGACTTCCAGCCAGTTGC‐3′, R: 5′‐TGCCATTGCACAACTCTTTTC‐3′), and β‐actin (F: 5′‐CCGCGAGTACAACCTTCTTG‐3′, R: 5′‐CGTCATCCATGGCGAACTGG‐3′).

### 2.11 Western Blot

Total proteins were extracted from the spinal cord tissues and rat astrocytes using radioimmunoprecipitation assay lysis buffer (Beyotime Biotechnology). Protein samples (equal amounts) were loaded onto SDS‐PAGE gels and transferred to PVDF membranes (Merck Millipore, USA). After blocking in 5% non‐fat milk for 1 h, membranes were incubated with primary antibodies against MAFG, C3, CRYAB, Serping1, DNMT3a (Abcam); S100A10, TNF‐α, IL‐1β (Proteintech); Sphk1, IL‐6, DNMT3b (Santa Cruz Biotechnology, USA); β‐actin and DNMT1 (Cell Signaling Technology, USA) overnight at 4°C, followed by incubation with the secondary antibodies (Abcam) for 1 h. To measure the optical density of protein bands, ImageJ software (NIH, USA) was used.

### 2.12 Enzyme‐Linked Immunosorbent Assay (ELISA)

After the indicated treatments, the supernatant of rat astrocytes was collected by centrifuging at 2000 *g* for 10 min. In the homogenate of the spinal cord tissues and supernatant of cells, the levels of IL‐6, IL‐1β, and TNF‐α were surveyed using corresponding ELISA assay kits (Beyotime Biotechnology).

### 2.13 Bisulfite Sequencing PCR (BSP)

Genomic DNA was extracted from rat astrocytes using a genomic DNA extraction kit (Tiangen Biotech Co. LTD, China). An EpiTect Fast DNA Bisulfite Kit (Qiagen, Germany) was used to modify and purify the genomic DNA. The converted DNA was used for the PCR analysis. BSP primers were designed using Meth‐Primer software. PCR products were cloned into the pMD19‐T vector (Takara Bio, Shiga, Japan) and sequenced. The online website MSRcall (http://www.msrcall.com/MSRcalcalate.aspx) was used to analyze the CRYAB methylation levels.

### 2.14 Statistical Analysis

Statistical analysis was performed using GraphPad Prism software (version 7.0; USA). The unpaired Student's t‐test was used for two‐group comparisons. One‐way ANOVA followed by Tukey's post hoc test and two‑way ANOVA followed by Bonferroni's post hoc test were used for multiple‑group comparisons. Brown‑Forsythe and Bartlett's tests were used to assess the normality and variance homogeneity. All experimental data were expressed as mean ± standard deviation. Statistical significance was set at *p* < 0.05.

## 3 Results

### 3.1 Activation of A1 Astrocyte is Accompanied by MAFG Upregulation

**Figure 1:**
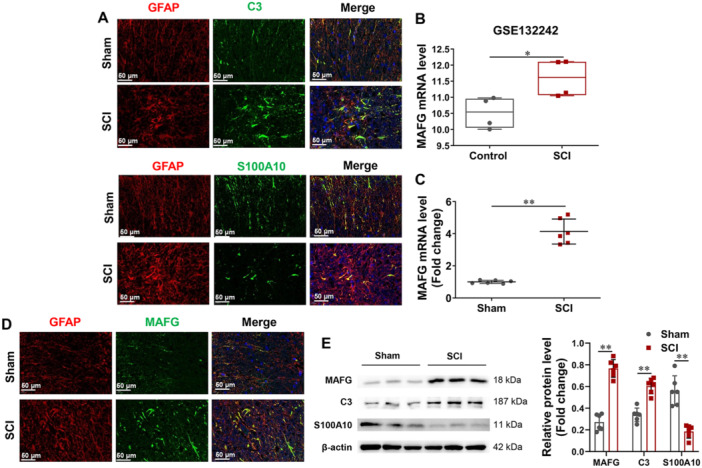
The activation of A1 astrocyte was accompanied by MAFG upregulation. (A) In spinal cord tissues of rats, C3‐ and S100A10‐positive cells were checked using immunofluorescence. (B) GSE132242 dataset showed that MAFG was overexpressed in mice after SCI (*n* = 4). In spinal cord tissues of rats, (C) RT‐PCR was applied to survey MAFG mRNA expression (*n* = 6); (D) MAFG‐positive cells were checked using immunofluorescence; (E) the protein expression of MAFG, C3, and S100A10 was surveyed through Western blot (*n* = 6). Data in (B, C, and E) were analyzed using the unpaired *t*‑test. *p < 0.05, **p < 0.01.

We used SD rats to construct an SCI animal model and detected changes in C3 (a marker of A1 reactive astrocytes) and S100A10 (a marker of A2 reactive astrocytes) in spinal cord tissues. GFAP was used to mark astrocytes. As shown in Figure 1A,E, compared with the sham group, C3 expression was increased and S100A10 expression was decreased in the spinal cord tissue of the SCI group, indicating that a large number of astrocytes transformed into type A1 astrocytes in the SCI rat spinal cord tissues. Based on the GSE132242 dataset, MAFG was significantly overexpressed in the SCI model compared to the control group (Figure 1B). Next, we assessed the changes in MAFG in the spinal cord tissue of rats using RT‐PCR, immunofluorescence, and Western blot analysis. Figure 1C–E showed that MAFG expression was increased in the spinal cord tissue of the SCI model compared to the control group.

### 3.2 Silencing of MAFG Improves Neurological Outcomes and Functional Recovery

**Figure 2:**
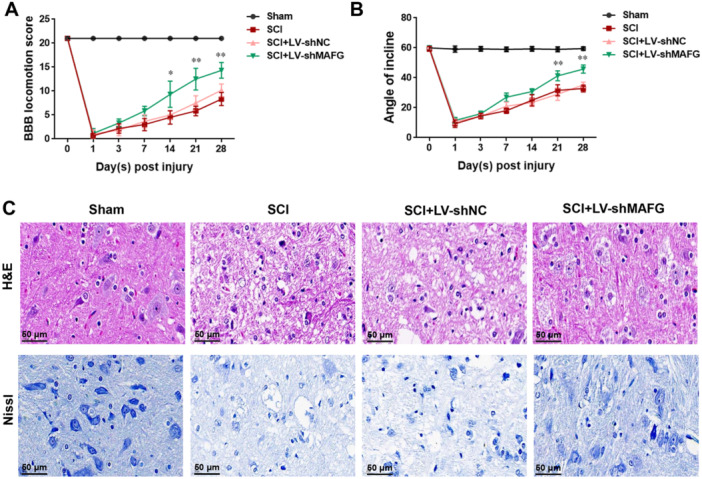
Silencing of MAFG improved neurological outcomes and functional recovery. (A) BBB scores were used to indicate the motor function of rats (*n* = 6). (B) The inclined plate test was carried out to evaluate the motor function of rats (*n* = 6). (C) H&E and Nissl staining were done to survey the pathological change in spinal tissue of rats. Data in (A and B) were analyzed using two‑way ANOVA followed by Bonferroni's post hoc test. *p < 0.05, **p < 0.01.

To investigate the influence of MAFG on SCI, LV‐shMAFG was injected into rats. Next, BBB scores were determined, and an inclined plate test was conducted. As shown in Figure 2A, in the sham group, the BBB score of the rats was 21, indicating no dyskinesia. In the SCI group, the BBB score of rats decreased significantly, and the BBB score was only 0.67 ± 0.52 on the first day after surgery; on the third day after surgery, the BBB score began to rise but remained low on the 28th day after surgery (8.33 ± 1.37). No significant difference was found for the BBB score of rats in SCI and SCI + LV‐shNC groups, but LV‐shMAFG enhanced the BBB score of rats on the 14th day after surgery compared with SCI + LV‐shNC group and raised the BBB score to 14.33 ± 1.63 on the 28th day after surgery. To further evaluate the motor function of rats, an inclined plate test was performed. Figure 2B shows that rats in the sham group remained on a 60° inclined plate for 5 s. However, rats in SCI group could only stay on the 9.33 ± 2.50° inclined plate for 5 s on the first day after surgery; from the third day after surgery, the test results of the rat's inclined plate improved, but the rat could only stay on 32.83 ± 2.32° inclined plate for 5 s on the 28th day after surgery. The results of the inclined plate test were not significantly different between the SCI and SCI + LV‐shNC groups. Compared with the SCI group, LV‐shMAFG significantly improved the test results of the inclined plate on the 21st day after surgery, and rats in the SCI + LV‐shMAFG could stay on the 45.83 ± 2.64° inclined plate for 5 s on the 28th day after surgery. These results indicated that silencing MAFG improved motor function in rats after SCI. Moreover, the rat spinal cord tissues were subjected to H&E and Nissl staining. Figure 2C shows that rats in the sham group had intact structures, whereas rats in the other three groups presented with damaged structural integrity and a decrease in the number of Nissl corpuscles in the spinal cord tissues. Importantly, compared to the SCI + LV‐shNC group, silencing of MAFG relieved the damaged structural integrity and the degree of nerve injury in rats after SCI.

### 3.3 Silencing of MAFG Inhibits the Activation of A1 Astrocyte and Neuroinflammation

**Figure 3:**
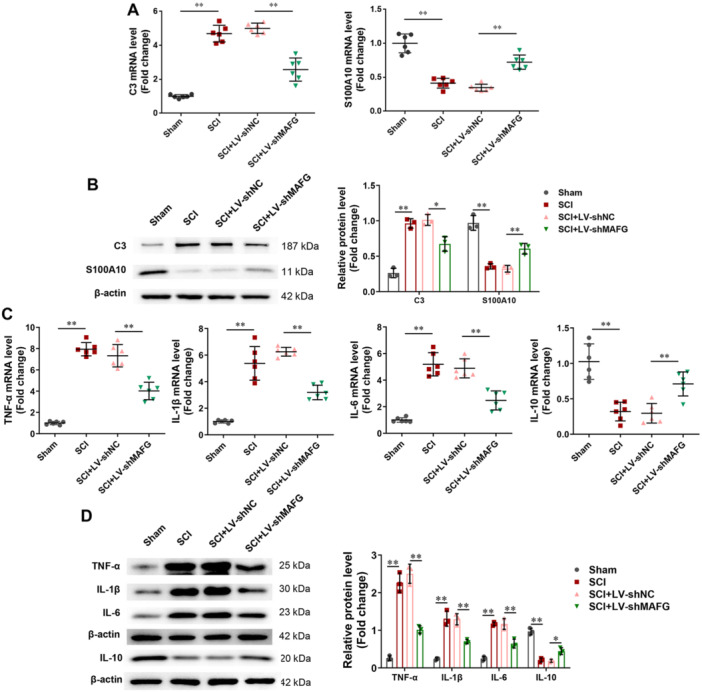
Silencing of MAFG inhibited the activation of A1 astrocyte and neuroinflammation. In spinal cord tissues of rats, (A) the mRNA expression of C3 and S100A10 was detected using RT‐PCR (*n* = 6); (B) the protein expression of C3 and S100A10 was detected using Western blot (*n* = 3); (C) the mRNA expression of TNF‐α, IL‐1β, IL‐6, and IL‐10 was detected using RT‐PCR (*n* = 6); (D) the protein expression of TNF‐α, IL‐1β, IL‐6, and IL‐10 was detected using Western blot (*n* = 3). All data were analyzed using one‑way ANOVA followed by Tukey's post hoc test. *p < 0.05, **p < 0.01.

Subsequently, we confirmed whether MAFG regulates the polarization of reactive astrocytes and inflammatory cytokines in vivo. Figure 3A,B showed that SCI increased C3 expression and decreased S100A10 expression in the spinal cord tissues of rats, whereas silencing of MAFG reversed these changes. Additionally, the results of RT‐PCR indicated that SCI increased the levels of pro‐inflammatory cytokines (TNF‐α, IL‐1β, and IL‐6) and decreased the level of anti‐inflammatory cytokine IL‐10 in spinal cord tissues of rats, but silencing of MAFG reduced the production of the pro‐inflammatory cytokines and increased the production of the anti‐inflammatory cytokine (Figure 3C). Similarly, the RT‐PCR results were verified by Western blotting (Figure 3D).

### 3.4 MAFG is Highly Expressed in the Activated A1 Astrocyte

**Figure 4:**
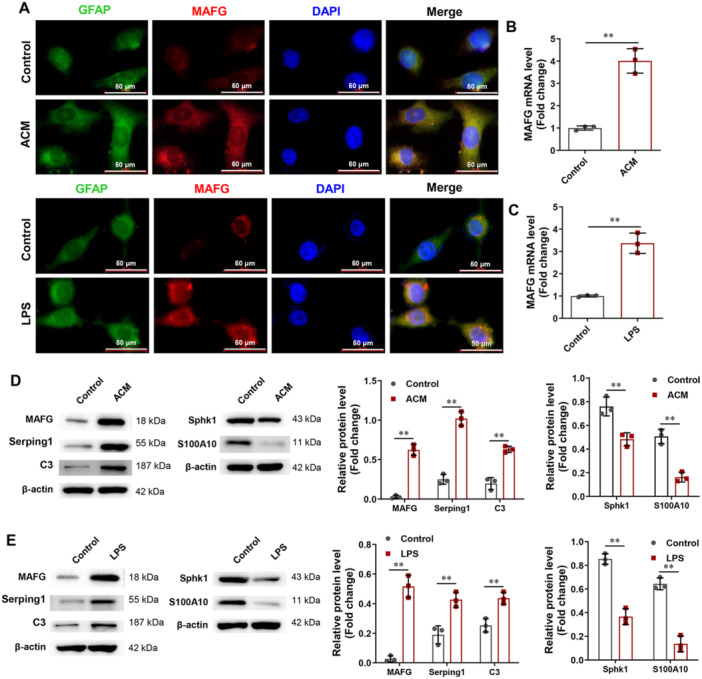
MAFG was highly expressed in the activated A1 astrocyte. (A) In rat astrocytes, MAFG positive cells were checked using immunofluorescence. (B and C) In rat astrocytes, MAFG mRNA expression was detected by RT‐PCR (*n* = 3). (D and E) In rat astrocytes, the protein expression of MAFG, Serping1, C3, Sphk1, and S100A10 was detected using Western blot (*n* = 3). Data in (B, C, D, and E) were analyzed using the unpaired *t*‑test. **p < 0.01.

To determine the function of MAFG in the conversion of A1 astrocytes, we isolated and cultured primary rat astrocytes and induced the generation of type A1 astrocytes using ACM or LPS. As shown in Figure 4A, ACM and LPS treatments increased the proportion of MAFG‐positive cells. Similarly, RT‐PCR and Western blotting showed that ACM and LPS promoted MAFG expression in rat astrocytes (Figure 4B–E). Moreover, the protein expression of Serping1 and C3 (markers of A1 reactive astrocytes) was enhanced by ACM and LPS, while the protein expression of Sphk1 and S100A10 (markers of A2 reactive astrocytes) was reduced by ACM and LPS (Figure 4D,E). These data suggested that ACM and LPS induced the generation of type A1 astrocytes and increased MAFG expression.

### 3.5 Silencing of MAFG Inhibits the Activation of A1 Astrocyte and Neuroinflammation in Rat Astrocytes

**Figure 5:**
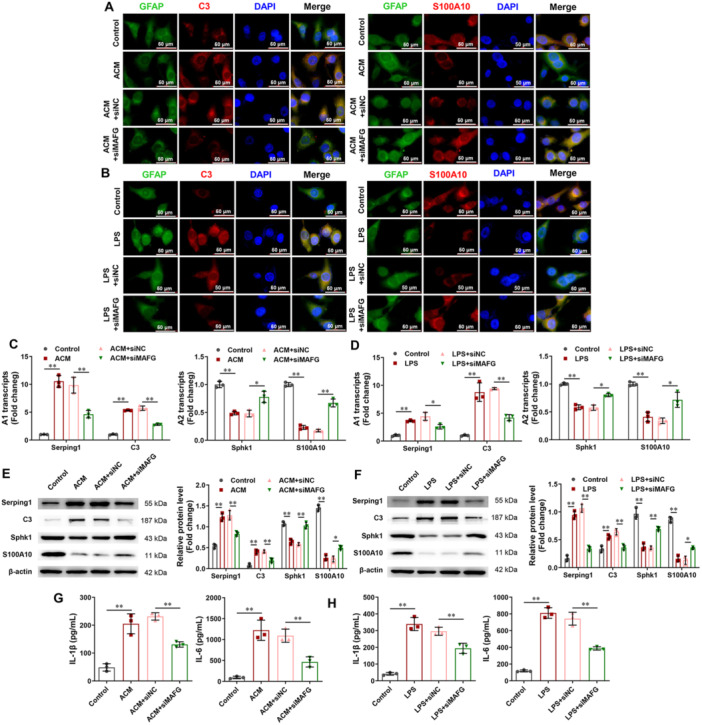
Silencing of MAFG inhibited the activation of A1 astrocyte and neuroinflammation in rat astrocytes. (A and B) In rat astrocytes, the positive cells of C3 and S100A10 were checked using immunofluorescence. (C and D) In rat astrocytes, the mRNA expression of Serping1, C3, Sphk1, and S100A10 were checked using RT‐PCR (*n* = 3). (E and F) In rat astrocytes, the protein expression of Serping1, C3, Sphk1, and S100A10 were checked using Western blot (*n* = 3). (G and H) In supernatant of rat astrocytes, the levels of IL‐1β and IL‐6 were examined by ELISA (*n* = 3). Data in (C, D, E, F, G, and H) were analyzed using one‑way ANOVA followed by Tukey's post hoc test. *p < 0.05, **p < 0.01.

We transfected rat astrocytes with siNC or siMAFG to further investigate the function of MAFG in regulating the polarization of reactive astrocytes and inflammatory cytokines in vitro. As shown in Figure 5A,B, ACM and LPS increased the proportion of C3‐positive cells and reduced the proportion of S100A10‐positive cells, whereas MAFG silencing decreased the proportion of C3‐positive cells and increased the proportion of S100A10‐positive cells. In addition, RT‐PCR and Western blotting revealed that ACM and LPS promoted Serping1 and C3 expression and inhibited Sphk1 and S100A10 expression, whereas silencing of MAFG inhibited Serping1 and C3 expression and promoted Sphk1 and S100A10 expression (Figure 5C–F). Figure 5G,H illustrate that ACM and LPS enhanced the levels of IL‐1β and IL‐6, while silencing of MAFG reduced the levels of IL‐1β and IL‐6.

### 3.6 Silencing of MAFG Inhibits the Methylation of CRYAB In Vitro

**Figure 6:**
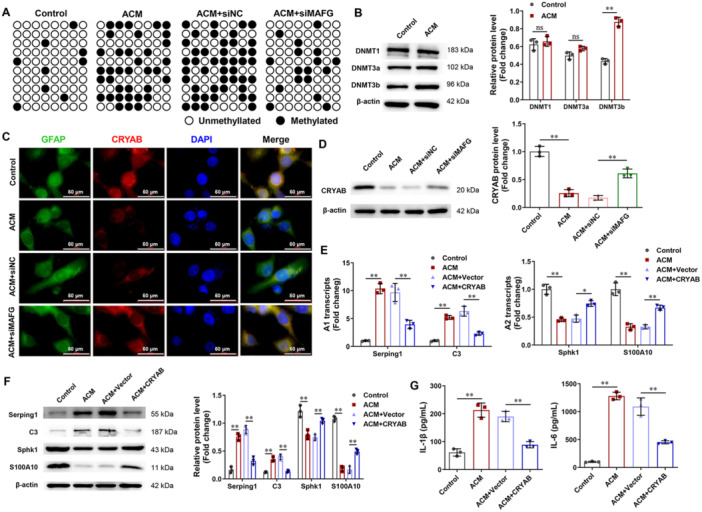
Silencing of MAFG inhibited the methylation of CRYAB in vitro which exerted anti‐inflammatory effects. (A) BSP was performed to detect CRYAB methylation (white represented unmethylation, black represented methylation). (B) In rat astrocytes, Western blot was applied to check the changes of key factors in DNA methylation (DNMT1, DNMT3a, and DNMT3b) (*n* = 3). (C) In rat astrocytes, the positive cells of CRYAB were checked using immunofluorescence. (D) In rat astrocytes, CRYAB protein expression was checked using Western blot (*n* = 3). (E) In rat astrocytes, the mRNA expression of Serping1, C3, Sphk1, and S100A10 was checked by RT‐PCR (*n* = 3). (F) In rat astrocytes, the protein expression of Serping1, C3, Sphk1, and S100A10 was checked using Western blot (*n* = 3). (G) In supernatant of rat astrocytes, the levels of IL‐1β and IL‐6 were examined by ELISA (*n* = 3). Data in (B, D, E, F, and G) were analyzed using one‑way ANOVA followed by Tukey's post hoc test. *p < 0.05, **p < 0.01.

To further explore the role of MAFG in astrocyte transformation, we used the BSP method to detect CRYAB methylation. Figure 6A shows that ACM promoted CRYAB methylation compared to the control group, whereas silencing of MAFG inhibited CRYAB methylation induced by ACM. Figure 6B shows that ACM promoted the expression of DNMT3b, rather than DNMT1 and DNMT3a, suggesting that the methylation of CRYAB in A1 reactive astrocytes might be catalyzed by DNMT3b. Furthermore, immunofluorescence and Western blotting showed that ACM inhibited CRYAB expression compared to the control group but silencing of MAFG increased CRYAB expression compared to the ACM+siNC group (Figure 6C,D).

To study the role of CRYAB in the transformation of A1 astrocytes, CRYAB overexpression astrocytes were transfected into rat astrocytes. Figures 6E,F show that CRYAB overexpression reduced the expression of Serping1 and C3 and increased the expression of Sphk1 and S100A10. Additionally, CRYAB overexpression reduced the levels of IL‐1β and IL‐6 in ACM‐treated rat astrocytes (Figure 6G).

### 3.7 Silencing of MAFG Inhibits the Activation of A1 Astrocyte and Neuroinflammation Via CRYAB Methylation

**Figure 7:**
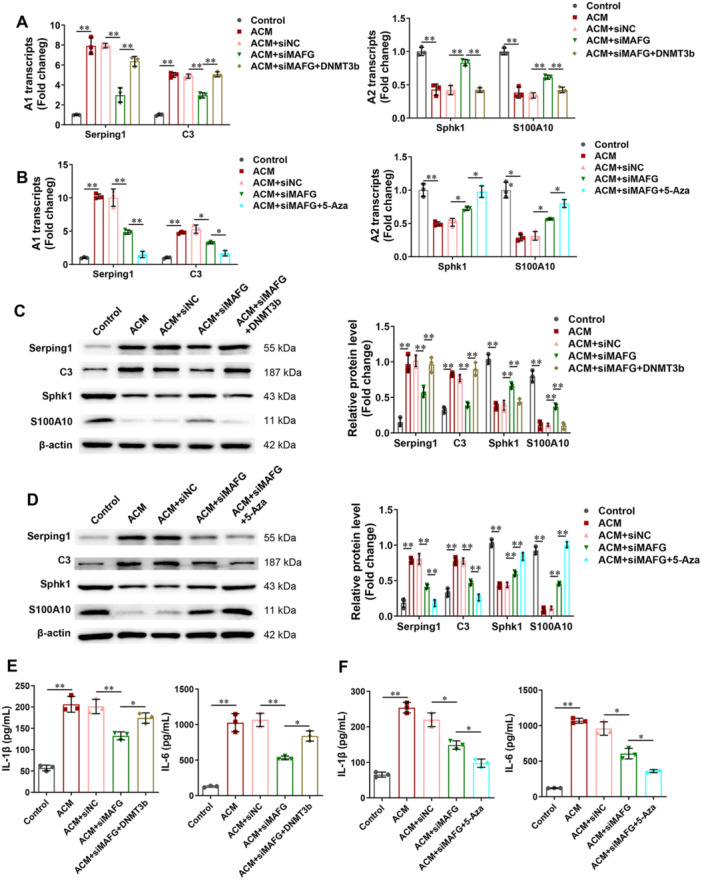
Silencing of MAFG inhibited the activation of A1 astrocyte and neuroinflammation via CRYAB methylation. (A and B) In rat astrocytes, the mRNA expression of Serping1, C3, Sphk1, and S100A10 was checked by RT‐PCR (*n* = 3). (C and D) In rat astrocytes, the protein expression of Serping1, C3, Sphk1, and S100A10 was checked using Western blot (*n* = 3). (E and F) In supernatant of rat astrocytes, the levels of IL‐1β and IL‐6 were examined by ELISA (*n* = 3). All data were analyzed using one‑way ANOVA followed by Tukey's post hoc test. *p < 0.05, **p < 0.01.

To verify the role of CRYAB methylation in MAFG, DNMT3b overexpression plasmid and siMAFG were co‐transfected into rat astrocytes. Figure 7A,C showed that DNMT3b overexpression reversed the inhibitory role of siMAFG on Serping1 and C3 expression and the promotive role of siMAFG on Sphk1 and S100A10 expression. In addition, 5‐Aza (an inhibitor of methylation) was used to treat rat astrocytes which were transfected with siMAFG. Figure 7B,D show that 5‐Aza further reduced Serping1 and C3 expression and increased Sphk1 and S100A10 expression in rat astrocytes transfected with siMAFG. Furthermore, compared with ACM+siMAFG group, DNMT3b overexpression reversed the inhibitory role of siMAFG on the levels of IL‐1β and IL‐6, and 5‐Aza further increased the inhibitory role of siMAFG on the levels of IL‐1β and IL‐6 (Figure 7E,F).

## 4 Discussion

SCI is a destructive neurological and pathological state that causes major motor, sensory and autonomic dysfunctions [1, 2]. Interventions for secondary injuries are important to improve tissue repair and functional recovery [28]. Research on secondary injuries has always drawn the interest of experts in rehabilitation physiotherapy, neurobiology, orthopedics, and neurosurgery; however, there is no widely accepted treatment plan for SCI [29, 30].

In the CNS, astrocytes are the most abundant cell population and play important roles in the formation and maintenance of the blood‐brain barrier, regulation of synaptogenesis, neurotransmitter recycling, and immune [31, 32, 33]. SCI can lead to astrocytic reactions, thus affecting functional outcomes [34]. A1 astrocytes produce neurotoxic factors, resulting in neuronal and oligodendrocyte apoptosis [35, 36]. In contrast, A2 astrocytes release neurotrophic factors and anti‐inflammatory cytokines, thereby exerting neuroprotective functions [37]. C3 is expressed only by A1 astrocytes and S100A10 is a specific marker of A2 astrocytes [32, 38]. At the lesion sites in the rat cord, C3 expression increased and S100A10 expression decreased. Importantly, in rat astrocytes treated with MCM and LPS, the expression trend of C3 and S100A10 was consistent with those observed *in vivo*. In addition, SCI models showed increased MAFG expression, indicating that MAFG may play an important role in SCI progression. To confirm the role of MAFG, LV‐shMAFG was used to infect rats with SCI. The results showed that silencing reduced C3 expression and enhanced S100A10 expression. Moreover, after SCI, silencing of MAFG promotes the recovery of motor function in rats, which might be related to a reduction in damaged structural integrity and the degree of nerve injury. Taken together, silencing MAFG improves neurological outcomes and functional recovery after SCI.

Central nervous system cells are activated by inflammatory cytokines [39]. Inflammatory cytokines have been found to promote and maintain inflammatory response [40]. In SCI, amplification of the inflammatory response is a key cause of excessive secondary injury [41]. Overexpression of pro‐inflammatory cytokines, such as TNF‐α, IL‐1β, and IL‐6, can promote apoptosis and result in the aggravation of nerve injury [42, 43, 44]. In this study, silencing of MAFG reduced the levels of TNF‐α, IL‐1β, and IL‐6 in SCI rats. Consistent with *in vivo* experiments, silencing of MAFG also reduced the levels of TNF‐α, IL‐1β, and IL‐6 in rat astrocytes treated with ACM or LPS. As expected, silencing MAFG inhibited neuroinflammation following SCI.

Recent studies have shown that CRYAB protects against ischemic stroke and intracerebral hemorrhage [45, 46]. In astrocytes, overexpression of CRYAB reduced pro‐inflammatory cytokines, including IL‐1β and IL‐6 [27]. Here, we studied the effect of MAFG on CRYAB expression and found that silencing MAFG inhibits CRYAB expression in SCI tissues. Furthermore, CRYAB overexpression reduced the levels of specific markers of A1 astrocytes (Serping and C3) and enhanced the levels of specific markers of A2 astrocytes (Sphk1 and S100A10). Additionally, overexpression of CRYAB reduced the production of TNF‐α, IL‐1β, and IL‐6 in ACM‐treated astrocytes. Taken together, these results indicate that CRYAB inhibits the activation of A1 astrocytes and neuroinflammation.

To further elucidate the role of MAFG in SCI, we examined the influence of MAFG on CRYAB methylation and found that silencing MAFG inhibited CRYAB methylation in ACM‐treated astrocytes. DNMT1, DNMT3a, and DNMT3b are key DNA methyltransferases that catalyze DNA methylation [47]. MAFG has been reported to cooperate with DNMT3B to limit gene expression [48]. In this study, ACM promoted DNMT3b expression, but not DNMT1 or DNMT3a expression. All these suggest that, MAFG cooperates with DNMT3b to induce CRYAB methylation and inhibit its gene expression in astrocytes. More importantly, the inhibitory effect of siMAFG on the activation of A1 astrocytes and the production of pro‐inflammatory cytokines were reversed by DNMT3b overexpression, whereas a methylation inhibitor (5‐Aza) further increased the inhibitory effect of siMAFG on the activation of A1 astrocytes and the production of pro‐inflammatory cytokines. Overall, silencing MAFG inhibited the activation of A1 astrocytes and neuroinflammation by regulating CRYAB methylation.

After SCI, MAFG expression was enhanced, and CRYAB expression was reduced in the injured spinal cord tissue of rats. *In vivo*, silencing of MAFG inhibited the activation of A1 astrocytes and neuroinflammation and promoted the recovery of motor function in rats. *In vitro*, in ACM‐treated rat astrocytes, silencing MAFG inhibited the activation of A1 astrocytes and the production of pro‐inflammatory cytokines related to CRYAB methylation.

## Author Contributions

Xuefei Li and Wanqing Qiao designed the study. Xuefei Li, Zhuang Zhu, Ying Wang, Hao Wan, Zhiwei Wang, and Wanqing Qiao performed the research and analyzed data. Xuefei Li and Wanqing Qiao wrote the article. All authors have read and approved the article.

## Ethics Statement

The experimental protocol of our study was performed in accordance with the Guide for the Care and Use of Laboratory Animals and approved by The Second Affiliated Hospital of Shandong First Medical University (2023‐A‐041).

## Consent

The authors have nothing to report.

## Conflicts of Interest

The authors declare no conflicts of interest.

## Data Availability

The data‐sets used and analyzed during the current study are available from the corresponding author on reasonable request.
